# Insertion of EGFP into the replicase gene of *Semliki Forest virus* results in a novel, genetically stable marker virus

**DOI:** 10.1099/vir.0.82436-0

**Published:** 2007-04

**Authors:** Nele Tamberg, Valeria Lulla, Rennos Fragkoudis, Aleksei Lulla, John K. Fazakerley, Andres Merits

**Affiliations:** 1 Estonian Biocentre, Tartu, Estonia; 2 Centre for Infectious Diseases, College of Medicine and Veterinary Medicine, University of Edinburgh, Edinburgh, UK; 3 Institute of Molecular and Cell Biology, University of Tartu, Tartu, Estonia

## Abstract

Alphavirus-based vector and replicon systems have been extensively used experimentally and are likely to be used in human and animal medicine. Whilst marker genes can be inserted easily under the control of a duplicated subgenomic promoter, these constructs are often genetically unstable. Here, a novel alphavirus construct is described in which an enhanced green fluorescent protein (EGFP) marker gene is inserted into the virus replicase open reading frame between nsP3 and nsP4, flanked by nsP2 protease-recognition sites. This construct has correct processing of the replicase polyprotein, produces viable virus and expresses detectable EGFP fluorescence upon infection of cultured cells and cells of the mouse brain. In comparison to parental virus, the marker virus has an approximately 1 h delay in virus RNA and infectious virus production. Passage of the marker virus *in vitro* and *in vivo* demonstrates good genetic stability. Insertion of different markers into this novel construct has potential for various applications.

Infectious cDNA (icDNA) clones and corresponding expression vectors have been developed for several alphaviruses (Liljestrom & Garoff, 1991; Strauss & Strauss, 1994; Simpson *et al.*, 1996). In most alphavirus-based expression vectors, the viral structural genes are replaced by foreign genes. These vectors are capable of only one round of infection and are thus non-mobilizable, a property that has severely hampered their use in studies that require a spreading, propagating infectious process. Marker and other recombinant alphaviruses have been produced by expressing the foreign gene from an internal ribosomal entry site element or from a duplicated 26S promoter (Raju & Huang, 1991; Hahn *et al.*, 1992; Pugachev *et al.*, 2000; Vaha-Koskela *et al.*, 2003). Double-subgenomic vectors containing marker genes allow direct observation of infection and have been used successfully in animal experiments (Levine *et al.*, 1996; Cook & Griffin, 2003). Unfortunately, these vectors tend to suffer from genome instability, probably because the inserted genes are introduced as separate transcription units and have no selective value (Pugachev *et al.*, 1995, 2000).

An alternative strategy is to insert genes into natural gene expression units. A marker gene has been inserted successfully into the Sindbis virus (SIN) structural gene (Thomas *et al.*, 2003). SIN genomes with markers inserted into the non-structural replicase to produce nsP3-fusion proteins have also been produced (Bick *et al.*, 2003; Frolova *et al.*, 2006). Production of non-structural–marker fusion proteins has also been successful in other viruses, including *Poliovirus*, *Hepatitis C virus*, *Equine arteritis virus* and several filamentous plant viruses (Mueller & Wimmer, 1998; Moradpour *et al.*, 2004; Rajamaki *et al.*, 2005; van den Born *et al.*, 2005). Here, we report that Semliki Forest virus (SFV) nsP3–enhanced green fluorescent protein (EGFP) fusion protein marker virus is viable, but genetically unstable; however, by the novel strategy of placing the EGFP insert between nsP3 and nsP4 flanked by duplications of the nsP3/4 nsP2 protease-recognition site, we have generated a viable, genetically stable virus with only minor changes in phenotype.

An SFV nsP3–EGFP fusion protein virus was engineered by subcloning and PCR-based mutagenesis. The C residue at position 5447 of pSFV4 (Liljestrom *et al.*, 1991) was changed to G and a 12 bp long sequence, GGGCCCATAGGATCC, was inserted after the modified codon. The resulting construct was designated pSFV(3F)4. The sequence encoding EGFP was PCR-amplified, cloned into pSFV(3F)4 and the resulting icDNA clone was designated pSFV(3F)4-EGFP (Fig. 1a[Fig f1]). Infectious virus, SFV(3F)4-EGFP, was obtained by electroporation of capped *in vitro* transcripts as described by Liljestrom *et al.* (1991). The primary virus stocks (P1) were collected after 24 h, titrated and used to infect fresh BHK-21 cells (m.o.i., 0.1). The second-passage stocks (P2) were also harvested at 24 h. SFV(3F)4-EGFP virus was viable and expressed the expected nsP3–EGFP fusion protein (Fig. 2a[Fig f2]). However, plaque purification followed by analysis of the fluorescence produced by individual plaques and Western blotting demonstrated that this virus was genetically unstable; a truncated form of the nsP3–EGFP fusion protein was present even in cells transfected directly with *in vitro*-synthesized RNA. The genetic instability of this virus is most probably due to defect(s) in the formation or functioning of replication complexes. As with SIN nsP3-fusion constructs (Ryman *et al.*, 2005; Frolova *et al.*, 2006; Ventoso *et al.*, 2006), SFV(3F)4-EGFP and its analogues may have uses to study replicase gene expression, replicase protein localization and interactions, but its instability precludes application in *in vivo* pathogenesis studies.

In a novel strategy, a second virus was engineered in which EGFP was placed between nsP3 and nsP4 flanked by nsP2 cleavage sites. Starting with pSFV(3F)4-EGFP, the C terminus of nsP3 was restored and an nsP2 protease-recognition site was added between nsP3 and EGFP (Fig. 1a[Fig f1]). The inserted protease-recognition sequence was based on the nsP3/4 junctional sequence, which is cleaved with high efficiency (Merits *et al.*, 2001; Vasiljeva *et al.*, 2001). The first amino acid residue after the inserted cleavage point was also changed from Tyr to Gly; nsP2 prefers Gly in the P1′ position and Gly represents a stabilizing amino acid (Lulla *et al.*, 2006; Varshavsky, 1996). The P1′ Gly was followed by aa 2–7 from the N terminus of nsP4 (Fig. 1a[Fig f1]). The region encoding the C terminus of nsP3 with indicated downstream sequence was created by PCR amplification and cloned into pSFV(3F)4-EGFP. The resulting clone and virus were designated pSFV(3H)4-EGFP and SFV(3H)4-EGFP, respectively (Fig. 1a[Fig f1]).

Infectious-centre assays (Gorchakov *et al.*, 2004) demonstrated that 3.1×10^5^ to 4.4×10^5^ plaques (μg transfected RNA)^−1^ were obtained for both pSFV(3H)4-EGFP and pSFV4 transcripts, with no statistically significant difference between them. In one-step growth studies (m.o.i., 20), relative to SFV4, production of infectious SFV(3H)4-EGFP was delayed by approximately 1 h (Fig. 1b[Fig f1]). However, as SFV(3H)4-EGFP was able to replicate to 10^9^ p.f.u. ml^−1^ within 10 h, it can be concluded that insertion of EGFP between nsP3 and nsP4 did not affect virus replication substantially.

To determine whether the EGFP insertion in SFV(3H)4-EGFP was inherited stably, 96 virus plaques were purified from each of five *in vitro* passages (P1–P5) in BHK-21 cells (m.o.i., 0.1) and each of five *in vivo* passages (M1–M5) in mouse brains. After each passage, the percentage of viruses expressing EGFP was assessed by random selection of 96 plaques followed by determination of EGFP expression. All plaques from the P2 stock of SFV(3H)4-EGFP were EGFP-positive and >90 % were EGFP-positive after the fifth passage. Even greater stability was observed for *in vivo*-propagated stocks: after the fifth passage, only one plaque (1/96) was EGFP-negative. As an EGFP-negative phenotype could result from EGFP inactivation by point mutation or by deletion, we RT-PCR-amplified and sequenced the corresponding regions for several of the EGFP-negative (*in vitro*-passaged) plaque-purified viruses. In all cases, these genomes contained large in-frame deletions, indicating that deletions in the marker gene do occur upon passage of SFV(3H)4-EGFP. Whilst the frequency of this process may be low, given their growth advantage, deleted genomes are likely to increase in the viral population following multiple passages.

In SFV-infected cells, nsP3 is known to associate with modified endosomes and lysosomes (Froshauer *et al.*, 1988). In contrast, free EGFP is typically localized diffusely in the cytoplasm and more abundantly in the nucleus. In SFV(3F)4-EGFP-infected BHK-21 cells, EGFP co-localized with nsP3 to punctate cytoplasmic structures, presumably virus replicase complexes (Fig. 1c[Fig f1]). In contrast, SFV(3H)4-EGFP-infected cells showed granular cytoplasmic staining for nsP3 and diffuse, predominantly nuclear staining for EGFP (Fig. 1d[Fig f1]). The independent localization of nsP3 and EGFP in the SFV(3H)4-EGFP-infected cells indicates that EGFP was released from the replication complexes.

To determine the phenotype of SFV(3H)4-EGFP *in vivo*, groups of six 5–6-week-old female BALB/c mice were inoculated (20 μl) intracerebrally with SFV4(3H)-EGFP or SFV4. All animal experiments were carried out under the authority of a UK Home Office licence and were approved by the University of Edinburgh ethical-review process. Mice in both groups had clinical signs of encephalitis at day 2 and were sampled. Half brains were fixed in 4 % neutral-buffered formalin for 16 h, processed through graded sucrose solutions, frozen in OCT and cut into sections (12 μm). EGFP-positive cells were observed readily in all (*n*=6) brains infected with SFV(3H)4-EGFP (Fig. 1e[Fig f1]). Titration of the other half brain, as described previously (Fazakerley *et al.*, 1993), demonstrated titres of infectious virus ranging from 1×10^9^ to 3×10^9^ p.f.u. g^−1^, with no difference between mice infected with SFV(3H)4-EGFP and those infected with parental SFV4. We conclude that, as with SFV4, SFV(3H)4-EGFP is neurovirulent and can replicate and spread efficiently in the mouse brain and that SFV(3H)4-EGFP-infected brain cells express sufficient EGFP to be observed readily by fluorescence microscopy.

Expression of the ns proteins in SFV(3H)4-EGFP- and SFV(3F)4-EGFP-infected BHK-21 cells was examined by Western blotting (Fig. 2a[Fig f2]). At 6 h post-infection, cells were lysed in Laemmli buffer and samples corresponding to 100 000 cells were separated by SDS-PAGE. Monospecific rabbit polyclonal antibodies were used to detect nsP1, nsP3 and nsP4, and a mouse monoclonal antibody was used to detect nsP2 (Peränen *et al.*, 1988; Laakkonen *et al.*, 1994; Rikkonen *et* *al.*, 1994; Kujala *et al.*, 1997). Individual nsP1, nsP2, nsP3, nsP4 and EGFP were observed readily in SFV(3H)4-EGFP-infected cells. No nsP3–EGFP fusion protein was detected, but a small amount of unprocessed EGFP–nsP4 was detected by antibodies to both nsP4 and EGFP. In SFV(3F)4-EGFP-infected cells, nsP3 was, as expected, present predominantly as an nsP3–EGFP fusion protein. A small amount of free nsP3 was also present; this may have resulted from the rapid generation of EGFP-negative genomes.

To study the dynamics of cleavage of the SFV(3H)4-EGFP polyprotein, infected cells (m.o.i., 100) were pulse labelled metabolically for 15 min with 50 μCi (1.85 MBq) [^35^S]methionine and [^35^S]cysteine, and nsP3 and its precursors were immunoprecipitated with antiserum against nsP3 and Protein A–Sepharose. Immunocomplexes were separated by SDS-PAGE and radiolabelled proteins were visualized by autoradiography. In pulse-labelled cells, the P123–EGFP, P3–EGFP–4 and P3–EGFP processing intermediates were all detected, whereas in chased samples, processing intermediates were not detected or were present in lower quantities, and the amount of fully processed nsP3 was increased (data not shown). Thus, all expected processing products, except the very rapidly processed P123–EGFP–4, were detected and no apparent disturbance of processing was observed.

In BHK-21 cells, EGFP is generally quite stable, with an estimated half-life of 24 h. However, in BHK-21 cells infected with SFV(3H)4-EGFP, the EGFP signal detected late in infection by fluorescence microscopy had decreased considerably, suggesting low EGFP stability. To assess the stability of the EGFP and ns proteins, BHK-21 cells infected with SFV(3H)4-EGFP or SFV4 were labelled metabolically, chased and EGFP, nsP1 and nsP3 were immunoprecipitated. For both viruses, only a small decrease in nsP1 and nsP3 was detected over 24 h (Fig. 2b[Fig f2]). In contrast, by 24 h, the amount of EGFP was below the detection limit of this analysis (Fig. 2b[Fig f2]). Thus, the low amounts of EGFP observed by microscopy indeed represent rapid degradation of EGFP. The processed EGFP has a Gly residue at its N terminus that, according the N-end rule, is stabilizing. Most likely, EGFP instability results from the duplicated C-terminal sequence of nsP3 (Fig. 1a[Fig f1]), which, as a result of the construction strategy, remains attached to the C terminus of EGFP. If this is the case, then the effect of this same sequence in its native location (C terminus of nsP3) is either different or is suppressed by interaction(s) with other components of the replicase complex.

Virus RNA synthesis by SFV4 and SFV(3H)4-EGFP was compared by [^3^H]uridine labelling. Relative to SFV4-infected cells, SFV(3H)4-EGFP-infected cells had a delay in virus RNA synthesis (Fig. 3a[Fig f3]); this was most obvious at 3–5 h post-infection and less so at later time points. To analyse whether insertion of the marker gene altered the temporal expression of SFV structural proteins or the shutdown of host-cell translation, protein synthesis in infected BHK-21 cells was studied by metabolic labelling. As observed with virus growth (Fig. 1b[Fig f1]) and RNA synthesis (Fig. 3a[Fig f3]), production of viral proteins C, E1 and p62 in SFV(3H)4-EGFP-infected cells started approximately 1 h later than in SFV4-infected cells. A longer, approximately 2 h, delay was observed for host-cell translational shutdown (Fig. 3b[Fig f3]).

In all measurements of virus replication and growth, SFV(3H)4-EGFP was slightly slower than SFV4. The possible reasons for this include effects resulting from larger ns polyprotein sizes and delays or defects in replicase-complex formation. However, these changes are unlikely to preclude use of this virus, as it replicates to high titres and remains neurovirulent. Despite the small duplication of viral sequence necessitated by the addition of the nsP2 processing site upstream of EGFP, SFV(3H)4-EGFP demonstrated remarkably improved genetic stability relative to SFV(3F)4-EGFP. However, it should be noted that, at late passages *in vitro*, some genomes with a deletion in the marker gene were present. Attempts to reduce this by removing the duplicated 6 aa from the N terminus of EGFP, or by decreasing the length of the nsP3 C-terminal fragment fused to the C terminus of EGFP, resulted in viable viruses, but did not increase their genetic stability (data not shown). Importantly, following replication and spread of SFV(3H)4-EGFP in the mouse brain, no deletion variants were observed until passage M5, demonstrating the utility of this virus for *in vivo* pathogenesis studies.

## Figures and Tables

**Fig. 1. f1:**
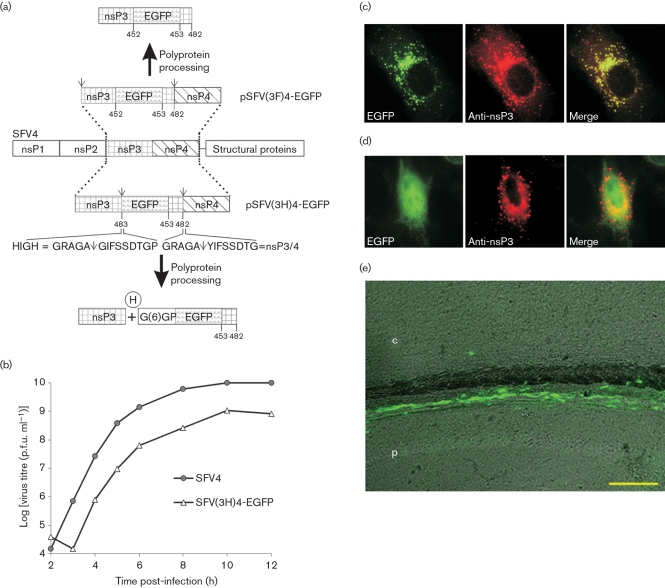
(a) Schematic representation of recombinant SFV genomes. The sequences of the inserted High (H) site and the native nsP3/4 nsP2 cleavage sites are shown. The 30 aa sequence from the C terminus of the nsP3 (aa 453–482), which was fused to the C terminus of EGFP, is indicated. The nsP2 protease-cleavage sites are indicated by ↓. Numbers indicate positions of amino acid residues in nsP3; (6) indicates the sequence IFSSDT (from nsP4). (b) Growth of SFV4 and SFV(3H)4-EGFP in BHK-21 cells infected with P2 viruses. (c) Localization of EGFP and nsP3 in BHK-21 cells infected with SFV(3F)4-EGFP. (d) Localization of EGFP and nsP3 in BHK-21 cells infected with SFV(3H)4-EGFP. In both (c) and (d), cells infected at an m.o.i. of 1 were fixed at 5 h post-infection. EGFP was detected by its fluorescence, and nsP3 with a polyclonal antibody and a secondary antibody conjugated to Texas red. (e) Virus-infected EGFP-positive cells (green) in mouse brain 2 days post-inoculation, detected by confocal microscopy; p denotes the hippocampal pyramidal cell layer and c the cortex. Bar, 100 μm.

**Fig. 2. f2:**
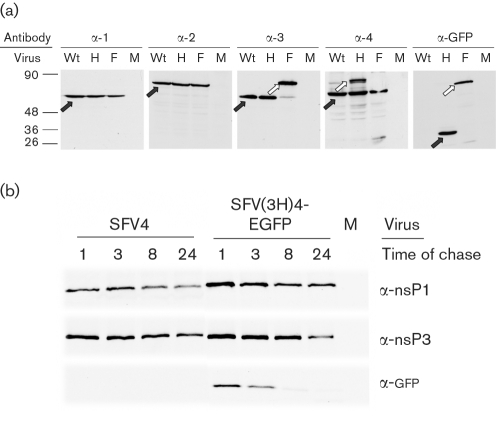
(a) Expression of EGFP and viral ns proteins by recombinant viruses. BHK-21 cells were infected (m.o.i., 20) with SFV4 (wt), P2 stock of SFV(3H)4-EGFP (H) or P1 stock of SFV(3F)4-EGFP (F), or were mock-infected (M), and cell lysates were analysed by Western blotting. The antibody used for detection is indicated at the top of each panel. Specific signals for nsP1, nsP2, nsP3, nsP4 or EGFP are indicated with a solid arrow; bands corresponding to nsP3–EGFP and EGFP–nsP4 fusion proteins are marked with an open arrow. (b) Proteins synthesized in infected cells were labelled metabolically at 3 h post-infection and chased for 1, 3, 8 or 24 h. Cell lysates were immunoprecipitated with anti-nsP1, anti-nsP3 or anti-EGFP antibodies and analysed by SDS-PAGE.

**Fig. 3. f3:**
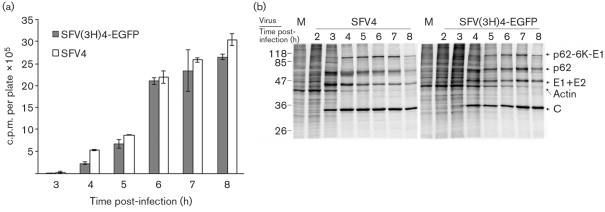
(a) Synthesis of virus RNA. BHK-21 cells were infected with SFV4 or SFV(3H)4-EGFP; at 1 h post-infection, actinomycin D (2 μg ml^−1^) and, at 2 h, 25 μCi (0.925 MBq) [^3^H]uridine, were added. Cells were collected at 3, 4, 5, 6, 7 and 8 h post-infection. Total RNA was precipitated by trichloroacetic acid. Incorporated radioactivity was measured by liquid scintillation. Each bar represents the mean of six replicates; error bars indicate sd. (b) Shutdown of cellular translation in BHK-21 cells infected with SFV4 or SFV(3H)4-EGFP. Proteins synthesized in infected cells were labelled metabolically at 1 h intervals and cell lysates were analysed by SDS-PAGE. M, Mock-infected cells.

## References

[r1] Bick, M. J., Carroll, J. W., Gao, G., Goff, S. P., Rice, C. M. & MacDonald, M. R. (2003). Expression of the zinc-finger antiviral protein inhibits alphavirus replication. J Virol 77, 11555–11562. 14557641 10.1128/JVI.77.21.11555-11562.2003 PMC229374

[r2] Cook, S. H. & Griffin, D. E. (2003). Luciferase imaging of a neurotropic viral infection in intact animals. J Virol 77, 5333–5338. 12692235 10.1128/JVI.77.9.5333-5338.2003 PMC153972

[r3] Fazakerley, J. K., Pathak, S., Scallan, M., Amor, S. & Dyson, H. (1993). Replication of the A7(74) strain of Semliki Forest virus is restricted in neurons. Virology 195, 627–637. 8393239 10.1006/viro.1993.1414

[r4] Frolova, E., Gorchakov, R., Garmashova, N., Atasheva, S., Vergara, L. A. & Frolov, I. (2006). Formation of nsP3-specific protein complexes during Sindbis virus replication. J Virol 80, 4122–4134. 16571828 10.1128/JVI.80.8.4122-4134.2006 PMC1440443

[r5] Froshauer, S., Kartenbeck, J. & Helenius, A. (1988). Alphavirus RNA replicase is located on the cytoplasmic surface of endosomes and lysosomes. J Cell Biol 107, 2075–2086. 2904446 10.1083/jcb.107.6.2075 PMC2115628

[r6] Gorchakov, R., Frolova, E., Williams, B. R., Rice, C. M. & Frolov, I. (2004). PKR-dependent and -independent mechanisms are involved in translational shutoff during Sindbis virus infection. J Virol 78, 8455–8467. 15280454 10.1128/JVI.78.16.8455-8467.2004 PMC479073

[r7] Hahn, C. S., Hahn, Y. S., Braciale, T. J. & Rice, C. M. (1992). Infectious Sindbis virus transient expression vectors for studying antigen processing and presentation. Proc Natl Acad Sci U S A 89, 2679–2683. 1372987 10.1073/pnas.89.7.2679 PMC48725

[r8] Kujala, P., Rikkonen, M., Ahola, T., Kelve, M., Saarma, M. & Kääriäinen, L. (1997). Monoclonal antibodies specific for Semliki Forest virus replicase protein nsP2. J Gen Virol 78, 343–351. 9018056 10.1099/0022-1317-78-2-343

[r9] Laakkonen, P., Hyvönen, M., Peränen, J. & Kääriäinen, L. (1994). Expression of Semliki Forest virus nsP1-specific methyltransferase in insect cells and in *Escherichia coli*. J Virol 68, 7418–7425. 7933125 10.1128/jvi.68.11.7418-7425.1994 PMC237184

[r10] Levine, B., Goldman, J. E., Jiang, H. H., Griffin, D. E. & Hardwick, J. M. (1996). Bc1-2 protects mice against fatal alphavirus encephalitis. Proc Natl Acad Sci U S A 93, 4810–4815. 8643485 10.1073/pnas.93.10.4810 PMC39361

[r11] Liljestrom, P. & Garoff, H. (1991). A new generation of animal cell expression vectors based on the Semliki Forest virus replicon. Biotechnology (N Y) 9, 1356–1361. 1370252 10.1038/nbt1291-1356

[r12] Liljestrom, P., Lusa, S., Huylebroeck, D. & Garoff, H. (1991). In vitro mutagenesis of a full-length cDNA clone of Semliki Forest virus: the small 6,000-molecular-weight membrane protein modulates virus release. J Virol 65, 4107–4113. 2072446 10.1128/jvi.65.8.4107-4113.1991 PMC248843

[r13] Lulla, A., Lulla, V., Tints, K., Ahola, T. & Merits, A. (2006). Molecular determinants of substrate specificity for Semliki Forest virus nonstructural protease. J Virol 80, 5413–5422. 16699022 10.1128/JVI.00229-06 PMC1472149

[r14] Merits, A., Vasiljeva, L., Ahola, T., Kääriäinen, L. & Auvinen, P. (2001). Proteolytic processing of Semliki Forest virus-specific non-structural polyprotein by nsP2 protease. J Gen Virol 82, 765–773. 11257180 10.1099/0022-1317-82-4-765

[r15] Moradpour, D., Evans, M. J., Gosert, R., Yuan, Z., Blum, H. E., Goff, S. P., Lindenbach, B. D. & Rice, C. M. (2004). Insertion of green fluorescent protein into nonstructural protein 5A allows direct visualization of functional hepatitis C virus replication complexes. J Virol 78, 7400–7409. 15220413 10.1128/JVI.78.14.7400-7409.2004 PMC434129

[r16] Mueller, S. & Wimmer, E. (1998). Expression of foreign proteins by poliovirus polyprotein fusion: analysis of genetic stability reveals rapid deletions and formation of cardioviruslike open reading frames. J Virol 72, 20–31. 9420196 10.1128/jvi.72.1.20-31.1998 PMC109345

[r17] Peränen, J., Takkinen, K., Kalkkinen, N. & Kääriäinen, L. (1988). Semliki Forest virus-specific non-structural protein nsP3 is a phosphoprotein. J Gen Virol 69, 2165–2178. 2970523 10.1099/0022-1317-69-9-2165

[r18] Pugachev, K. V., Mason, P. W., Shope, R. E. & Frey, T. K. (1995). Double-subgenomic Sindbis virus recombinants expressing immunogenic proteins of Japanese encephalitis virus induce significant protection in mice against lethal JEV infection. Virology 212, 587–594. 7571428 10.1006/viro.1995.1516

[r19] Pugachev, K. V., Tzeng, W. P. & Frey, T. K. (2000). Development of a rubella virus vaccine expression vector: use of a picornavirus internal ribosome entry site increases stability of expression. J Virol 74, 10811–10815. 11044128 10.1128/jvi.74.22.10811-10815.2000 PMC110958

[r20] Rajamaki, M. L., Kelloniemi, J., Alminaite, A., Kekarainen, T., Rabenstein, F. & Valkonen, J. P. (2005). A novel insertion site inside the potyvirus P1 cistron allows expression of heterologous proteins and suggests some P1 functions. Virology 342, 88–101. 16112702 10.1016/j.virol.2005.07.019

[r21] Raju, R. & Huang, H. V. (1991). Analysis of Sindbis virus promoter recognition in vivo, using novel vectors with two subgenomic mRNA promoters. J Virol 65, 2501–2510. 2016769 10.1128/jvi.65.5.2501-2510.1991 PMC240605

[r22] Rikkonen, M., Peränen, J. & Kääriäinen, L. (1994). ATPase and GTPase activities associated with Semliki Forest virus nonstructural protein nsP2. J Virol 68, 5804–5810. 8057461 10.1128/jvi.68.9.5804-5810.1994 PMC236984

[r23] Ryman, K. D., Meier, K. C., Nangle, E. M., Ragsdale, S. L., Korneeva, N. L., Rhoads, R. E., MacDonald, M. R. & Klimstra, W. B. (2005). Sindbis virus translation is inhibited by a PKR/RNase L-independent effector induced by alpha/beta interferon priming of dendritic cells. J Virol 79, 1487–1499. 15650175 10.1128/JVI.79.3.1487-1499.2005 PMC544143

[r24] Simpson, D. A., Davis, N. L., Lin, S. C., Russell, D. & Johnston, R. E. (1996). Complete nucleotide sequence and full-length cDNA clone of S.A.AR86 a South African alphavirus related to Sindbis. Virology 222, 464–469. 8806532 10.1006/viro.1996.0445

[r25] Strauss, J. H. & Strauss, E. G. (1994). The alphaviruses: gene expression, replication, and evolution. Microbiol Rev 58, 491–562. 7968923 10.1128/mr.58.3.491-562.1994 PMC372977

[r26] Thomas, J. M., Klimstra, W. B., Ryman, K. D. & Heidner, H. W. (2003). Sindbis virus vectors designed to express a foreign protein as a cleavable component of the viral structural polyprotein. J Virol 77, 5598–5606. 12719552 10.1128/JVI.77.10.5598-5606.2003 PMC154044

[r27] Vaha-Koskela, M. J., Tuittila, M. T., Nygardas, P. T., Nyman, J. K., Ehrengruber, M. U., Renggli, M. & Hinkkanen, A. E. (2003). A novel neurotropic expression vector based on the avirulent A7(74) strain of Semliki Forest virus. J Neurovirol 9, 1–15. 10.1080/13550280390173382 12587064

[r28] van den Born, E., Stein, D. A., Iversen, P. L. & Snijder, E. J. (2005). Antiviral activity of morpholino oligomers designed to block various aspects of *Equine arteritis virus* amplification in cell culture. J Gen Virol 86, 3081–3090. 16227231 10.1099/vir.0.81158-0

[r29] Varshavsky, A. (1996). The N-end rule: functions, mysteries, uses. Proc Natl Acad Sci U S A 93, 12142–12149. 8901547 10.1073/pnas.93.22.12142 PMC37957

[r30] Vasiljeva, L., Valmu, L., Kaariainen, L. & Merits, A. (2001). Site-specific protease activity of the carboxyl-terminal domain of Semliki Forest virus replicase protein nsP2. J Biol Chem 276, 30786–30793. 11410598 10.1074/jbc.M104786200

[r31] Ventoso, I., Sanz, M. A., Molina, S., Berlanga, J. J., Carrasco, L. & Esteban, M. (2006). Translational resistance of late alphavirus mRNA to eIF2alpha phosphorylation: a strategy to overcome the antiviral effect of protein kinase PKR. Genes Dev 20, 87–100. 16391235 10.1101/gad.357006 PMC1356103

